# State-Level Policy Environments, Discrimination, and Victimization among Sexual and Gender Minority People

**DOI:** 10.3390/ijerph19169916

**Published:** 2022-08-11

**Authors:** Kristen D. Clark, Mitchell R. Lunn, Eliot M. Lev, Michael A. Trujillo, Micah E. Lubensky, Matthew R. Capriotti, Thomas J. Hoffmann, Juno Obedin-Maliver, Annesa Flentje

**Affiliations:** 1 Department of Nursing, University of New Hampshire, Durham, NH 03824, USA; 2 The PRIDE Study/PRIDEnet, Stanford University School of Medicine, Stanford, CA 94304, USA; 3 Division of Nephrology, Department of Medicine, Stanford University School of Medicine, Stanford, CA 94304, USA; 4 Department of Epidemiology and Population Health, Stanford University School of Medicine, Stanford, CA 94304, USA; 5 Department of Community Health Systems, University of California San Francisco School of Nursing, San Francisco, CA 94143, USA; 6 Department of Psychology, Carnegie Mellon University, Pittsburgh, PA 15213, USA; 7 Department of Psychology, San Jose State University, San Jose, CA 95192, USA; 8 Department of Epidemiology & Biostatistics, University of California San Francisco School of Medicine San Francisco, San Francisco, CA 94143, USA; 9 Department of Obstetrics and Gynecology, Stanford University School of Medicine, Stanford, CA 94304, USA; 10 Alliance Health Project, Department of Psychiatry, School of Medicine, University of California, San Francisco, CA 94143, USA

**Keywords:** policy, sexual and gender minority, LGBT, stigma, discrimination, victimization

## Abstract

Legislation has been passed in some states to reduce discrimination and victimization toward sexual and gender minority people (SGM; people who are not solely heterosexual and/or whose gender identity is not equal to what is socially associated with sex assigned at birth). The purpose of these analyses is to test whether state-level policy environments are associated with past-year discrimination and victimization among SGM people. Cross-sectional data from The Population Research in Identity and Disparities for Equality (PRIDE) Study annual questionnaire (collected 2018–2019), a national study of the health of SGM adults in the USA, were used for these analyses. Measures included related to discrimination, victimization, and demographic characteristics. State-level policy environments were measured using data from the Movement Advancement Project. Logistic regression analyses evaluated state-level policy environment scores and past-year discrimination and victimization among gender identity categories. In this sample, 7044 people (gender minority *n* = 2530) were included. Cisgender sexual minority (odds ratio [OR] = 1.007, *p* = 0.041) and the gender expansive subgroup of gender minority people (OR = 1.010, *p* = 0.047) in states with more protective policy environments had greater odds of discrimination. The gender expansive subgroup was found to have greater odds of victimization in states with more protective policy environments (OR = 1.003, *p* < 0.05). There was no relationship between state-level policy environments and victimization among any other study groups. SGM people may experience increased risk for discrimination and victimization despite legislative protections, posing continued risks for poor health outcomes and marginalization. Evaluation of factors (e.g., implementation strategies, systems of accountability) that influence the effectiveness of state-level polices on the reported experiences of discrimination and victimization among SGM people is needed.

## 1. Introduction

Sexual and gender minority (SGM) people experience disparities in physical and mental health outcomes, including high rates of depressive symptoms, elevated rates of substance use, and obesity [1,2,3,4]. For the purpose of this paper, sexual minority refers to people whose sexual orientation is not heterosexual (e.g., people who are lesbian, gay, bisexual, or another sexual orientation). Gender minority refers to people whose gender identity is not the same as that which is traditionally associated with one’s sex assigned at birth, such as transgender or non-binary people. Discrimination and victimization based on sexual orientation and/or gender identity contribute to SGM health disparities due to the excess stress from these and other harmful experiences (i.e., minority stress) [5,6]. Government policies that explicitly include sexual orientation and gender identity as protected classes are a structural intervention to reduce the discrimination and victimization experienced by SGM people.

Anti-discrimination and hate crime policies that criminalize discrimination and victimization toward SGM people exist in the United States of America (USA) at the federal and state levels and are associated with changes in the reporting of certain types of discrimination and victimization events. Same-sex marriage recognition is correlated with a reduction in reported hate crimes among sexual minority (SM) people [7]. State-level anti-bullying policies and other SGM protections are associated with less bullying and cyberbullying [8]. Another study found that gender minority (GM) people perceived less community stigma in states with anti-discrimination laws [9]. These findings suggest that state-level nondiscrimination policies result in less stigma toward SGM people, particularly a reduction in reported discrimination and victimization. However, the relationships between policies, policy environments, and discrimination and victimization among SGM people are increasingly ambiguous. For example, a study on state policies as a moderator to minority stress and suicide attempts among GM people found that the low policy protection states were associated with greater suicide attempts but found no other differences among moderate or high policy protection states [10]. State-level healthcare policy protections were found to have no moderating effect in the relationship between past-healthcare mistreatment and healthcare avoidance among GM people [11]. Another study that examined legal marriage recognition by the United States Supreme Court’s decision on the Defense of Marriage Act (Obergefell v. Hodges) found that the ruling coincided with a temporary increase in discrimination or victimization from people who endorsed conservative beliefs directed toward sexual minority people [12]. However, federal policies, such as hate crimes that expressly include sexual orientation and gender identity as protected classes, are enforced by federal authorities. Unless individual states include sexual orientation and gender identity explicitly as protected classes in anti-discrimination and hate crime policies, there is no mandated state enforcement of these protections and states are not required to report them to federal authorities [13]. Therefore, the relevance of these state-level policies on the experiences of SGM people is distinct.

The social ecological model describes the relationship between levels of society and the experiences of the individual. At the structural level, a state-level policy environment may be protective toward SGM people. With these laws in place, people in the community where an SGM person resides may be less likely to engage in stigmatizing behaviors, such as discrimination or acts of violence, resulting in fewer reports of these events [14,15]. At the community level, there have been observations of a “societal backlash” toward a marginalized group who has gained protections. This phenomenon has been observed at the state (or structural) level toward racial and ethnic minority people [16]. A national study of GM people found that state-level policies prohibiting employment discrimination based on gender identity were associated with greater odds of unemployment [17]. A more recent study on the attitudes of the general public toward SGM people found that 40% of American adults opposed marriage equality and supported the rights of private businesses to discriminate against SGM people [18], suggesting that there may be a rise in negative attitudes toward SGM people in these years following the Obergefell v. Hodges ruling that legalized marriage equality in the USA. Changes in attitudes may not be readily indicated at the structural level, as equal marriage protections are still in place despite a rise in anti-SGM attitudes. However, additional examination is necessary to understand if state-level policy environments have similar negative consequences or a “societal backlash” on SGM people’s interpersonal experiences.

While the noted studies evaluate state-level policy environments and the experiences of SGM people, the current literature does not evaluate subgroups of SGM people (e.g., cisgender sexual minority people and subgroups of GM individuals, such as transfeminine people). Furthermore, the association between state-level policies around sexual orientation and gender identity and reports of discrimination and victimization among gender expansive people (a subgroup of GM people who do not solely describe their gender identity or expression as man or woman) is unknown. Examining the relationship between SGM-related protective policy environments and experiences of gender expansive GM people is particularly needed since gender expansive GM people may be more vulnerable to discrimination or victimization [19,20].

This study seeks to understand whether state-level policy environments, measured through Movement Advancement Project [21] policy scores, are associated with reported past-year discrimination and victimization among SGM people. We hypothesize that states with greater protections will be associated with lower odds of reporting past-year discrimination and victimization. Additionally, this study aims to identify whether recent (i.e., within one year prior) changes in state-level policy environments are related to reports of past-year discrimination and victimization among SGM people during the following year. We hypothesize that recent increases in state-level policy environment scores will be associated with an increase in reported past-year discrimination and victimization during the following year, indicating an increase in state-level societal stigma toward SGM people. Since many policies only include a portion of SGM communities (e.g., solely protect people based on sexual orientation or lack representation of diverse gender identities), we looked at these relationships separately among cisgender SM people, binary GM people of any sexual orientation (i.e., solely transmasculine or transfeminine), and with gender expansive GM people of any sexual orientation.

## 2. Materials and Methods

Data were collected within the 2018 Annual Questionnaire of The PRIDE Study, a national, longitudinal, online, cohort study of SGM people who reside in the USA. The PRIDE Study is an online community-engaged research study with an active Participant Advisory Committee that reviewed all measures for SGM inclusivity and reviewed the study described here. Recruitment for The PRIDE Study was conducted through PRIDEnet (a national LGBTQ+ community engagement network), community partners, online communications (e.g., blog posts, newsletters, advertising on social media), in-person outreach at conferences and events, the distribution of The PRIDE Study promotional items, and word-of-mouth (see [22,23] for detailed description of The PRIDE Study).

Prior to enrollment in The PRIDE Study, participants were prompted with a webpage that described the study and allowed them to provide informed consent. Eligible individuals were 18 years or older, lived in the USA or its territories, could read and understand English, and had an SGM identity. Participants access surveys on their dashboard and receive notifications for the Annual Questionnaires and other surveys that they may complete. Enrolled participants who completed the 2018 Annual Questionnaire measures outlined in these analyses between June 2018 and May 2019 were included in the sample. State-level policy environments, or the quantification of how protective or unprotective a state’s environment is, was measured through data provided by the Movement Advancement Project [21] and extracted on 8 October 2019. These data were merged with The PRIDE Study data based on participants state of residence.

### 2.1. Measurement

Participant demographics included age, race/ethnicity, sexual orientation, gender identity, sex assigned at birth, highest education level completed, and household gross income. State of residence was determined through participant-provided ZIP code. Age was calculated by subtracting participants’ birth date (obtained upon study enrollment) from the date that the survey was started. Race and ethnicity were measured by a categorical variable where participants could select all options that apply: American Indian or Alaska native, Asian, Black, Hispanic/Latino/Spanish, Middle Eastern or North African, Native Hawaiian or other Pacific Islander, white, and “none of these fully describe me” with a free text response box. Sexual orientation was measured by a categorical variable that asked participants “What is your current sexual orientation?”. Participants could select all of the following options that apply: asexual, bisexual, gay, lesbian, pansexual, queer, questioning, same-gender loving, straight/heterosexual, and “another sexual orientation” with a free text response box.

Gender identity was measured by a categorial variable that asked participants “what is your current gender identity?” Participants could select all of the following options that apply: genderqueer, man, transgender man, transgender woman, woman, and “another gender identity” with a free text response box. Sex assigned at birth was measured with a variable that asked, “What was your sex assigned at birth, for example on your original birth certificate?” Participants could respond female or male.

Participants were categorized as cisgender SM if they endorsed a sexual orientation that was not solely “straight/heterosexual” and endorsed a gender identity traditionally associated with the sex that they were assigned at birth (e.g., endorsed gender of man and sex assigned at birth of male). Participants were categorized as having a binary GM identity if they endorsed a gender identity that was on a solely masculine or solely feminine spectrum and different from that traditionally associated with sex that they were assigned at birth, including people of any sexual orientation as in Flentje et al. [24]. This encompassed participants who, for example, endorsed (1) transgender man or another masculine gender identity (e.g., masculine non-binary, demiboy) and were assigned a female sex at birth and (2) transgender woman or another feminine gender identity (e.g., demigirl, non-binary femme) and were assigned male at birth. Participants were categorized as the gender expansive GM subgroup if they endorsed a non-binary gender identity (e.g., genderqueer, genderfluid) or genders that were both masculine and feminine (e.g., transmasculine and woman) or neither masculine nor feminine (e.g., agender). These gender identity categories included people of any sexual orientation.

Highest education level was measured by an ordinal variable with 10 options ranging from “no schooling” to “professional degree”. We coded this in our analyses as a 4-level variable (i.e., “no high school diploma”, “high school/GED graduate or some college”, “college degree [2- or 4-year]”, and “graduate degree”.) Household income was measured by an ordinal 11-item variable ranging from USD 0 to USD 100,000+. Demographic variables included in both The PRIDE Study, and these analyses, are provided in Table 1 as measured in the survey, except for household income which was collapsed for brevity.

State-Level policy environments were measured with data from the Movement Advancement Project. The Movement Advancement Project reports each state’s SGM-inclusive legislation to create a state-level policy environment score [21]. For example, a state with employment anti-discrimination laws that explicitly apply to both SM and GM people received 2 out of 2 points, whereas a state without employment anti-discrimination laws for either SM or GM people would receive 0 out of 2 points. If a state were to have no protections in any of the items and only harmful policies (e.g., laws banning cities from passing anti-discrimination laws, religious exemption laws), the state could have a negative score. The possible scores for each state range from −17.5 to 35. This policy environment score was included as a single continuous variable. While separate scores could be obtained for policies related to sexual orientation and gender identity, we retained a single continuous variable score encompassing both policies for each state. This is because our GM sample (*n* = 2530) is almost 97% SM people. Therefore, sexual orientation policies are important to the GM sample’s experiences of discrimination and victimization. Additionally, previous research shows that attitudes toward GM people are predictive of attitudes toward cisgender SM people [25,26,27,28]; therefore, the gender identity polices are relevant for the cisgender SM sample’s experiences of discrimination and victimization.

Recent changes in state-level policy environments were measured using data from the Movement Advancement Project. The state-level policy environment scores that were published by the Movement Advancement Project twelve months prior to participant data collection were subtracted from scores used to represent the policy environment at the time of participant data collection. This provided a new variable that indicated the change in state-level policy environment scores (sample range: −2 to 9.25), reflecting a recent change in policy environment. Positive numbers indicated an increase in state-level policy environment protectiveness (i.e., more protections for SGM people); zero indicated no change; negative numbers indicated a decrease in the protective state-level policy environment (i.e., a reduction in protections for SGM people). This variable was included as a single continuous variable in our analysis.

Participant reports of discrimination and victimization were measured using nine items about specific types of experienced discrimination (i.e., in employment, housing, when receiving services, in educational settings, in medical settings, by law enforcement, harassment from strangers) and types of violent victimization (i.e., physical attacks or injuries or unwanted sexual contact) that occurred within the past 12 months. For each type of past-year discrimination and victimization, participants could indicate yes or no. If yes was selected, participants could indicate the reason they believe that they were discriminated against or victimized by selecting all the following options that applied: dis/ability status, age, body composition, gender identity, gender expression, race and/or ethnicity, sexual orientation, or “something else”. These items were derived from the National HIV Behavioral Surveillance [29] surveys and were expanded and adapted based on expert review, participant advisory committee review, and participant feedback. The items were recoded to a dichotomous variable where past-year discrimination that was attributed to their gender identity, gender expression, or sexual orientation was coded as 1. A second dichotomous variable using the same procedure was used for the victimization outcome.

### 2.2. Analysis

Participants without data for state were dropped (*n* = 236, 3.4%). Descriptive statistics are reported for demographic variables and individual measures of discrimination and victimization among the three study groups: cisgender SM people and two subgroups of GM people, binary GM people of any sexual orientation and gender expansive GM people of any sexual orientation. For our primary analysis within each of the 3 study groups (cisgender SM, binary GM, and gender expansive GM), we conducted separate logistic regression analyses to test the two dependent variables, past-year discrimination and past-year victimization. We tested the independent variable, state-level policy scores, in one model and then fit a separate model to test the independent variable, change in state-level policy scores. We evaluated the linear fit of the independent variables and included a quadratic term for state-level policy scores and past-year victimization. All models controlled for age, race, ethnicity, education level, and household income. While sexual orientation was a variable used to inform the study groups, we included this as a covariate because differences in discrimination and victimization have been observed among sexual minority subgroups [30,31]. As a sensitivity analysis, we examined model fit with a random effect for state to account for unmeasured characteristics by state that could be related to our independent or dependent variables. The results from the sensitivity analysis are provided in the Supplementary Materials Tables S1 and S2.

As a secondary analysis, we tested for within-group differences. Within-group differences by gender or sex assigned at birth were analyzed using interaction terms for each of the three population groups (i.e., cisgender SM women verses cisgender SM men, transfeminine versus transmasculine people, and gender expansive GM people assigned female at birth versus gender expansive GM people assigned male at birth). Within-group difference by education, race/ethnicity, and age were analyzed using interaction terms for each of the three population groups (i.e., college/university degree versus less than college/university degree, white versus another race/ethnicity, and age). As these analyses were secondary to primary study aims, a Bonferroni correction was used to reduce type I error; significance for these interactions was set at *p* < 0.001 [32]. All analyses were run using Stata 15 [33].

## 3. Results

### 3.1. Participants

Participant characteristics are presented in Table 1. A total of 7044 participants were included in this analysis; 64.1% were cisgender SM people, 12.6% were binary GM people of any sexual orientation, and 23.5% were gender expansive GM people of any sexual orientation. The mean age of participants was 34.50 years (SD = 13.24), and the sample was predominantly white and non-Hispanic, with 81.61% describing themselves as solely white. Nearly three-quarters (74.54%) of participants had earned a college degree, and 31.92% reported a household income of more than USD 60,000 annually.

### 3.2. State-Level Policy and Reported Past-Year Discrimination

The state-level policy environment scores in relation to reported past-year discrimination are presented in Table 2. Greater state-level policy environment scores were associated with a greater odds of reporting past-year discrimination among cisgender SM people (odds ratio [OR] = 1.007; 95% confidence interval [CI] = 1.000–1.015; *p* = 0.041) and the gender expansive GM subgroup of any sexual orientation (OR = 1.010; 95% CI = 1.000–1.022; *p* = 0.047). This indicates that, in our sample, state-level policy environments that were more protective were associated with slightly greater odds of discrimination during the past year among these two population groups. The model covarying for state random effect found the same relationships (SM people, OR = 1.010; 95% CI = 1.000–1.029; *p* = 0.040; gender expansive GM subgroup (OR = 1.010; 95% CI = 1.00–1.020; *p* = 0.042; Supplementary Materials Table S1). There was no relationship between state-level policy environment scores and reported past-year discrimination reported by the binary GM subgroup of any sexual orientation. Interactions examining within group differences by gender or sex assigned at birth, education, race/ethnicity, and age were not statistically significant.

### 3.3. State-Level Policy and Reported Past-Year Victimization

We examined the state-level policy environment scores and reported past-year victimization (Table 2). Greater state-level policy environment scores were associated with a greater odds of reporting past-year victimization among the gender expansive GM subgroup (OR = 1.003; 95% confidence interval [CI] = 1.000–1.026; *p* = 0.040). There was no relationship between state-level policy environment scores and reported past-year victimization among any other group. Models that covaried for state random effects did not converge (Supplementary Materials Table S1).

### 3.4. Recent Changes in State-Level Policy Environments and Reported Past-Year Discrimination and Victimization

We examined the recent change in state-level policy environment and reported past-year discrimination and victimization (Table 3). There was no relationship between recent changes in state-level policy environment scores and reported past-year discrimination or victimization among any group, nor in the models that covaried for state random effects (Supplementary Materials Table S2).

## 4. Discussion

This study sought to determine whether state-level policy environments are related to reported past-year discrimination and victimization reported by SGM people. We found that greater state-level policy environment scores (indicating a more protective environment) were associated with greater odds of reporting past-year discrimination, but not victimization, among cisgender SM people. Among the gender expansive GM subgroup, greater state-level policy environment scores were associated with greater odds of reporting both past-year discrimination and victimization. However, state-level policy environment scores were not related to reported past-year discrimination or victimization among the binary GM subgroup. While our effect sizes in these analyses were small (range: 1.003–1.010), relationships that affect large numbers of people, as in the case of SGM people in a given state, are meaningful in terms of population health [34]. Importantly, policy protections were not related to less experiences of discrimination or victimization in any of our analyses.

Changes in state-level protections between 2017 and 2019 were not associated with reported past-year discrimination or victimization among any of our analysis groups. Legal institutions, such as state legislatures, can play a significant role in reducing acts of harm (i.e., discrimination and victimization) directed toward marginalized groups [35]. However, attitudes underpinning those acts of harm can be slow to change, particularly when they pertain to historically contentious issues, such as stigmatized social identities. Another consideration is the variation in implementation and enforcement of the policy protections. Where implementation strategies are well-planned and robust, greater reduction in mistreatment has been observed [36]. Implementation should include efforts to educate and inform the protected group of their rights and recourse. However, there is distrust in institutions as policies are not always implemented consistently or at all [37,38]. Recently in the USA, gender-affirming care for GM youth has been deemed child abuse in the state of Texas; however, prosecutors in some municipalities have stated their refusal to pursue parents who seek affirming healthcare for their children [39]. This has occurred in several areas of the USA related the recent rollback of abortion rights in which some municipalities are refusing to pursue legal action against those who seek abortion care in their jurisdictions [40]. While these are examples of the rights of individuals being protected by lack of enforcement, the inverse also occurs. Therefore, the relationship between changes in policies and protections is complex and requires deeper investigation than what we are unable to conduct given the limitations of our data.

While previous studies found positive outcomes where state-level policies were protective, such as reduced suicidal ideation or attempts [10,41,42] or greater utilization of healthcare services [43], we found either null results or greater odds of discrimination and victimization. The social context in the USA, as viewed through the social ecological model, is an important consideration to interpret these unexpected findings. The activism and leadership of SGM people and allies in the USA at the community level led to a watershed of SGM-protective policies implemented between 2008 and 2016 at the federal level and in many states, which resulted in more protective policy environments at the structural level [44,45]. Since 2016, there has been considerable sociopolitical change in the USA, subsequently changing the policy environments for SGM people. These changes have been especially visible as related to GM people where state-level policies targeting the civil liberties of this group have risen sharply [46]. This is an example of the reciprocal relationship between community-level attitudes and structural-level policies Evidence of these impacts can be observed at the community level as increases in federally reported hate crimes have occurred during the past several years (2017–2022) [47,48,49,50] and increased experiences of rejection and stigma from broader society have been reported at the community level by non-SGM people and individual level by SGM people [18,38,51]. Another possible explanation for our findings is that the broadened conversation and acknowledgement of discrimination and victimization toward SGM people that occurs in the community level and accompanies the passage of state-level policy protections at the structural level may validate individual experiences among cisgender SM people and gender expansive GM people, heighten awareness, and subsequently increase reporting; this is a phenomenon observed in other social movements [52]. In contrast, a person who experiences discrimination but has no protections against these experiences may be more likely to ignore, or not report, these experiences due to their lack of control or recourse in the situation. Thus, denying or minimizing these experiences may serve to exert control over one’s environment or as a means of coping with negative experiences [53].

We found greater past-year reports of overall discrimination and victimization as state-level policy environment scores increased among the gender expansive GM subgroup. Whether policy protections explicitly protect GM people who do not exist as solely masculine or feminine, such as gender expansive GM people, is an important consideration to this finding. Societal attitudes toward GM people remain predominantly based on a perception of gender as a binary construct [54], where gender expansive GM people who may be perceived as outside of that gender binary may be at greater risk of stigma. Furthermore, state-level policies where gender is assumed to be a solely binary construct may render legal protections ambiguous in their application to gender expansive GM people, thereby leaving them more vulnerable to mistreatment and violence [19,20,55]. This can be most fundamentally observed through the process of changing identification documents, where, in most states, individuals must still choose “male” or “female”. Therefore, the existence of a policy intended to provide protections based on gender identity may be less effective for those whose gender is assumed to be solely man or solely woman.

The reports of discrimination by SGM individuals and a shift toward acknowledgement of those experiences by the dominant societal group generally precedes state-level policy changes [56]. Policies are one mechanism for addressing the harmful experiences reported by marginalized groups. Policy environment scores are one way to quantify societal stigma toward SGM people. However, additional time may be necessary for these changes to subsequently be observed in day-to-day experiences and for longer term social change to occur [56]. This process may take more time in communities where heterosexism or cisgenderism are more common and socially accepted [57]. Those communities may be more resistant to changes that protect marginalized groups, in this case changes in policy environments for SGM people. While we cannot determine the differences between communities within a specific state due to the lack of community-level data in our sample and in the MAP measurement, these differences could underlie our findings among cisgender SM people and gender expansive GM people in our sample.

The minority stress model posits that harmful health outcomes can be explained by additional stress experienced by SGM people due to their marginalized sexual orientation and/or gender identity [6,19]. Our findings show that more protective policy environments are associated with greater reports of past-year discrimination and victimization among cisgender SM people and gender expansive GM people. Discrimination is associated with a wide range of disparities, including poor mental [58,59] and physical health outcomes [60,61] and with delays in seeking healthcare services [11,62]. Further research should examine whether policy environments moderate the relationship between reported discrimination and victimization and the health of SGM people. The empowerment of SGM people through more protective state-level polices may lessen the potential health implications of discrimination and victimization.

There are several limitations to this work. First, that the sample was limited in racial and ethnic diversity; this limits the generalizability of the findings to racial and ethnic minority groups and increases concerns of overall sampling bias. For example, our study reflects participants with higher levels of education and income levels than what has been found in more representative samples [63], which likely impacts the experiences of participants in our sample and the subsequent findings. Further, recruitment of participants to The PRIDE Study involves event participation and community organization engagement. While other recruitment strategies are in place, such as internet ads, and individuals often choose to participate for numerous other reasons (e.g., to contribute to the health and wellbeing of their communities), there could be bias toward participants who are more community-connected and have heightened awareness of the experiences of peers and advocacy happening related to SGM rights. Replication of these analyses with a representative sample, including SGM people from rural areas where fewer community supports are available, is needed. Our measurement of discrimination was limited to nine types of discrimination, but few examples were provided to participants in the survey. This may result in a lack of clarity and possible under-measurement of past-year experiences. Further, the limited yes/no response options for discrimination and victimization items do not allow us to account for multiple experiences of the same type or the severity of that event. Some types of discrimination or victimization may be occurring at greater frequency or differences in severity that we could not analyze in relationship with state-level policy environments. For example, participants could be experiencing less verbal harassment than prior to the existence of a policy, but this would be measured the same with a yes/no answer choice. We are unable to capture this nuance. As part of our discrimination and victimization dependent variable, we only include instances where the event occurred due to sexual orientation, gender identity, or gender expression. This requires participants to make judgements based on their perceptions and increases opportunity for error due to misattribution. We were unable to obtain MAP state-level policy environment scores that matched the exact window of time that The PRIDE Study’s Annual Questionnaire responses were obtained as participants could respond anytime between June 2018 and June 2019. Since we could not extract unique policy scores for each timepoint that participants answered the survey, a cut-off point was chosen. During this gap, individual policy changes may have occurred that are not reflected in our analysis. Related to measurement of state-policy environments, future work should examine the differences in the experiences among GM people who live in states with few GM protections and greater SM protections. Further, our analysis only examined state-level policy environments, not community or municipality policies and associated experiences. This is important because many municipalities have passed policy protections for SGM people that are not present at the state level; therefore, the experiences of individuals in a city may vary from individuals in that same state but a different location [64]. The use of MAP scores as a continuous measure is also a limitation since each unit is not necessarily uniform, nor is it equal between and among states. Future work including the development of validated measures of policy or social environments [65,66] would advance our understanding of the level of social stigma and how it relates to reports of discrimination and victimization among SGM people. Further, our findings could be related to individual characteristics within certain states or characteristics of the states themselves (e.g., neighboring state policies on SGM protections [67], types of policies implemented [21], differences in characteristics of the policies [68]), all of which are unaccounted for in our analyses. Future work should evaluate the relationship between policy environments and other state-level factors, such as religiosity or political party state legislature control. Another consideration is that perpetrators of violence are not always aware of the legal protections related to sexual orientation and gender identity, which could impact our findings.

## 5. Conclusions

Our study found that more protective state-level policy environments are associated with greater odds of reported discrimination and victimization among both cisgender, SM people and gender expansive GM people. It is imperative for public health professionals, community advocates, and other stakeholders to advocate for increased support and resources for SGM people, even within protective policy environments. Researchers should ask SGM individuals whether and how experiences of discrimination and victimization have changed in the presence of state policies rather than assuming/looking for benefits from improvements to the policy environment. Further work aimed at examining the mechanisms underlying our observations is needed to better understand how individuals in more protective policy environments perceive and experience stigma and how these experiences may affect their health.

## Figures and Tables

**Table 1:** Characteristics of The PRIDE Study 2018 Annual Questionnaire Participants (*N* = 7044).

Variable	Total Sample (*N* = 7044)	Cisgender Sexual Minority (*n* = 4514)	Binary Gender Minority ^a^ (*n* = 877)	Gender Expansive ^a^ (*n* = 1653)
Personal characteristics				
Age, in years (Mean ± SD)	34.50, 13.24	36.47, 13.87	33.87, 13.15	29.48, 9.67
Race/ethnicity ^b^				
American Indian or Alaska Native	207 (2.94)	1056 (2.33)	29 (3.31)	73 (4.42)
Asian	331 (4.70)	201 (4.45)	34 (3.88)	96 (5.81)
Black, African American, or African	241 (3.42)	152 (3.37)	31 (3.53)	58 (3.51)
Hispanic, Latino, or Spanish	463 (6.57)	324 (7.18)	46 (5.25)	93 (5.63)
Middle Eastern or North African	105 (1.49)	65 (1.44)	9 (1.03)	31 (1.88)
White	6398 (90.83)	4071 (90.19)	809 (92.25)	1518 (91.83)
Another race/ethnicity than is listed	147 (2.09)	73 (1.04)	17 (0.24)	57 (0.81)
Gender Identity				
Another Gender Identity Only	350 (4.97)	5 (0.11)	10 (1.14)	335 (20.27)
Genderqueer Only	407 (5.78)	0 (0)	0 (0)	407 (24.62)
Man Only	1984 (28.17)	1947 (43.13)	37 (4.22)	0 (0)
More than 1 gender identity	1271 (18.04)	15 (0.33) ^c^	351 (39.4)	911 (55.11)
Transgender Man Only	287 (4.07)	0 (0)	287 (32.73)	0 (0)
Transgender Woman Only	171 (2.43)	0 (0)	171 (19.50)	0 (0)
Woman Only	2574 (36.54)	2547 (56.42)	27 (3.08)	0 (0)
Sexual Orientation				
Asexual Only	142 (2.03)	63 (1.41)	26 (3.03)	53 (3.23)
Bisexual Only	688 (9.86)	514 (11.47)	83 (9.67)	91 (5.55)
Gay Only	1611 (23.09)	1508 (33.66)	68 (7.93)	35 (2.13)
Lesbian Only	858 (12.30)	699 (15.60)	73 (8.51)	86 (5.24)
Pansexual Only	243 (3.48)	97 (2.17)	77 (8.97)	69 (4.21)
Queer Only	541 (7.75)	173 (3.86)	106 (12.35)	262 (15.98)
Questioning Only	17 (0.24)	5 (0.11)	9 (1.05)	3 (0.18)
Straight/Heterosexual Only	79 (1.13)	0 (0.00)	76 (8.86)	3 (0.18)
Another Sexual Orientation Only	35 (0.50)	9 (0.20)	6 (0.70)	20 (1.22)
More than 1 Sexual Orientation	2764 (39.61)	1412 (31.52)	334 (38.93)	1018 (62.07)
Socioeconomic position				
Annual household income				
<USD 20 K	634 (10.53)	312 (8.17)	94 (12.24)	228 (15.90)
USD 20 K to <USD 40 K	1102 (18.31)	591 (15.48)	170 (22.14)	341 (23.78)
USD 40 K to <USD 60 K	1014 (16.85)	611 (16.01)	140 (18.23)	263 (18.34)
USD 60 K to <USD 80 K	794 (13.19)	509 (13.34)	116 (15.10)	169 (11.79)
≥USD 80 K	2475 (41.12)	1794 (47.00)	248 (32.29)	433 (30.20)
Educational level				
No high school diploma	46 (0.75)	16 (0.41)	12 (1.53)	18 (1.22)
High school/GED graduate or some college	1522 (24.71)	748 (19.15)	288 (36.73)	486 (33.04)
College degree (2- or 4-year)	2410 (39.12)	1527 (39.10)	299 (38.14)	584 (39.70)
Graduate degree	2182 (35.42)	1614 (41.33)	185 (23.60)	383 (26.04)

Notes: The number of participants in the study group with available data are reported as (*n*) and percent (%) of *n* for each variable. ^a^ Gender minority participants include participants of any sexual orientation. ^b^ Category is not mutually exclusive; therefore, percentages may be greater than 100%. ^c^ Cisgender participants with >1 gender identity included participants who endorsed a gender that solely lies on a spectrum that aligns with the sex they were assigned at birth (e.g., woman, femme, and assigned female at birth). Abbreviations: General Education Development (GED), Standard deviation = SD.

**Table 2:** Results of logistic regression models examining the relationship between state-level policy environment scores and past-year discrimination and victimization within The PRIDE Study (*N* = 7044).

Dependent Variable Predicted by State-Level Policy Scores (OR, *p*, CI)	Total Sample (*N* = 7044)	Cisgender, Sexual Minority (*n* = 4514)	Binary Gender Minority (*n* = 877)	Gender Expansive (*n* = 1653)
Discrimination Experiences	**OR = 1.009, *p* < 0.01**	**OR = 1.007, *p* = 0.041**	OR = 1.0091, *p* = 0.263	**OR = 1.010, *p* = 0.047**
	**95% CI (1.004–1.014)**	**95% CI (1.000–1.015)**	95% CI (0.993–1.026)	**95% CI (1.000–1.022)**
Victimization Experiences	OR = 0.950, *p* = 0.096	OR = 0.968, *p* = 0.428	OR = 1.031, *p* = 0.821	**OR = 0.902, *p* = 0.043**
	95% CI (0.895–1.002)	95% CI (0.892–1.050)	95% CI (0.790–1.346)	**95% CI (0.816–0.997)**
Victimization Experiences (quadratic term)	OR = 1.002, *p* = 0.071	OR = 1.001, *p* = 0.339	OR = 1.000, *p* = 0.906	**OR = 1.003, *p* = 0.040**
	95% CI (1.000–1.003)	95% CI (0.999–1.003)	95% CI (0.992–1.008)	**95% CI (1.000–1.026)**

All models covaried for age, race, ethnicity, sexual orientation, education level, and household income. Abbreviations: Total sample (*N*), Sample subgroup (*n*), Odds ratio (OR), Confidence Interval (CI). Bolded results are statistically significant (*p* < 0.05).

**Table 3:** Results of logistic regression analyses for changes in state-level policy scores (2017–2019) and their association with past year discrimination and victimization among an online national cohort of sexual and/or gender minority people (*N* = 7044).

Dependent Variable (OR, *p*, CI)	Total Sample (*N* = 7044)	Cisgender, Sexual Minority (*n* = 4514)	Binary Gender Minority (*n* = 877)	Gender Expansive (*n* = 1653)
Discrimination Experiences	OR = 0.994, *p* = 0.664	OR = 0.985, *p* = 0.415	OR = 0.989 *p* = 0.784	OR = 1.007, *p* = 0.770
	95% CI (0.969–1.020)	95% CI (0.950–1.021)	95% CI (0.912–1.072)	95% CI (0.963–1.052)
Victimization Experiences	OR = 1.016, *p* = 0.815	OR = 1.028, *p* = 0.760	OR = 1.363, *p* = 0.266	OR = 0.922, *p* = 0.478
	95% CI (0.889–1.161)	95% CI (0.860–1.230)	95% CI (0.790–2.354)	95% CI (0.737–1.154)
Victimization Experiences (quadratic term)	OR = 0.995, *p* = 0.599	OR = 0.997, *p* = 0.760	OR = 0.950, *p* = 0.287	OR = 1.003, *p* = 0.835
	95% CI (0.975–1.015)	95% CI (0.860–1.024)	95% CI (0.864–1.044)	95% CI (0.971–1.037)

All models covaried for age, race, ethnicity, sexual orientation, education level, and household income. Abbreviations: Odds ratio (OR), Confidence Interval (CI). Bolded results are statistically significant (*p* < 0.05).

## Data Availability

Due to the agreements with The PRIDE Study participants and the participant advisory committee, we are not able to make data publicly available. Data is available by request through submission of an ancillary study application with The PRIDE Study.

## Supplementary Materials

The following supporting information can be downloaded at: https://www.mdpi.com/article/10.3390/ijerph19169916/s1, Table S1: Results of logistic regression models examining the relationship between state-level policy environment scores and past-year discrimination and victimization within The PRIDE Study (*N* = 7044) with a random effect for state; Table S2: Results of logistic regression analyses for changes in state-level policy scores (2017–2019) and their association with past year discrimination and victimization among an online national cohort of sexual and/or gender minority people (*N* = 7044) with a random effect for state.
