# Supplementary material for: State-Level Policy Environments, Discrimination, and Victimization among Sexual and Gender Minority People
Source: Int J Environ Res Public Health. 2022 Aug 11;19(16):9916. doi: 10.3390/ijerph19169916 (PMC9407724; doi:10.3390/ijerph19169916)
Supplement: Supplementary file 1 [file ijerph-19-09916-s001.zip › ijerph-1788267-supplementary.pdf]

Supplementary Table S1. Results of logistic regression models examining the relationship between state-level policy environment scores and past-year discrimination and victimization within The PRIDE Study ($N = 7044$) with a random effect for state.

Dependent Variable predicted by state-level policy scores *(OR, p, CI)*	Cisgender, Sexual Minority ($n = 4514$)	Binary Gender Minority ($n = 877$)	Gender Expansive ($n = 1653$)
**Discrimination Experiences**	**OR = 1.010, *p* = 0.040 95% CI (1. 000–1.029)**	Not Converged	**OR = 1.010, *p* = 0.042 95% CI (1.000–1.020)**
**Victimization Experiences**	Not Converged	Not Converged	Not Converged

All models covaried for age, race, ethnicity, sexual orientation, education level, and household income.
Abbreviations: Odds ratio (OR), Confidence Interval (CI)
Bolded results are statistically significant ($p < 0.05$).

Supplementary Table S2. Results of logistic regression analyses for changes in state-level policy scores (2017–2019) and their association with past year discrimination and victimization among an online national cohort of sexual and/or gender minority people ($N = 7044$) with a random effect for state.

Dependent Variable predicted by state-level policy scores *(OR, p, CI)*	Cisgender, Sexual Minority ($n = 4570$)	Binary Gender Minority ($n = 889$)	Gender Expansive ($n = 1685$)
**Discrimination Experiences**	OR = 0.995, $p = 0.824$ 95% CI (0.953–1.039)	OR = 1.004 $p = 0.924$ 95% CI (0.919–1.080)	OR = 1.006, $p = 0.816$ 95% CI (0.956–1.058877)
**Victimization Experiences**	Not Converged	Not Converged	OR = 0.958, $p = .806$ 95% CI (0.680–1.350)

All models covaried for age, race, ethnicity, sexual orientation, education level, and household income.
Abbreviations: Odds ratio (OR), Confidence Interval (CI)
Bolded results are statistically significant ($p < 0.05$).
